# Genetic polymorphisms in key hypoxia-regulated downstream molecules and phenotypic correlation in prostate cancer

**DOI:** 10.1186/s12894-017-0201-y

**Published:** 2017-01-31

**Authors:** Avelino Fraga, Ricardo Ribeiro, André Coelho, José Ramon Vizcaíno, Helena Coutinho, José Manuel Lopes, Paulo Príncipe, Carlos Lobato, Carlos Lopes, Rui Medeiros

**Affiliations:** 1 Department of Urology, Porto Hospital Centre – St. António Hospital, Largo Prof. Abel Salazar, 4000-001 Porto, Portugal; 2 Center for Urological Research, Department of Urology, Porto Hospital Centre – St. António Hospital, Porto, Portugal; 3 ICBAS, Abel Salazar Biomedical Sciences Institute, University of Porto, Porto, Portugal; 4 Molecular Oncology Group - CI, Portuguese Institute of Oncology, Porto, Portugal; 5 Genetics Laboratory, Faculty of Medicine, University of Lisbon, Lisbon, Portugal; 6 Department of Pathology, Porto Hospital Centre – St. António Hospital, Porto, Portugal; 7 Department of Pathology and Oncology, Faculty of Medicine, University of Porto, Porto, Portugal; 8 Institute of Pathology and Molecular Immunology of University of Porto (IPATIMUP), Porto, Portugal; 9 Department of Urology, Porto Military Hospital, Porto, Portugal

**Keywords:** Genetic polymorphism, Hypoxia, Hypoxia-inducible factor 1, Prostate cancer

## Abstract

**Background:**

In this study we sought if, in their quest to handle hypoxia, prostate tumors express target hypoxia-associated molecules and their correlation with putative functional genetic polymorphisms.

**Methods:**

Representative areas of prostate carcinoma (*n* = 51) and of nodular prostate hyperplasia (*n* = 20) were analysed for hypoxia-inducible factor 1 alpha (HIF-1α), carbonic anhydrase IX (CAIX), lysyl oxidase (LOX) and vascular endothelial growth factor (VEGFR2) immunohistochemistry expression using a tissue microarray. DNA was isolated from peripheral blood and used to genotype functional polymorphisms at the corresponding genes (*HIF1A* +1772 C > T, rs11549465; *CA9* + 201 A > G; rs2071676; *LOX* +473 G > A, rs1800449; *KDR* – 604 T > C, rs2071559).

**Results:**

Immunohistochemistry analyses disclosed predominance of positive CAIX and VEGFR2 expression in epithelial cells of prostate carcinomas compared to nodular prostate hyperplasia (*P* = 0.043 and *P* = 0.035, respectively). In addition, the VEGFR2 expression score in prostate epithelial cells was higher in organ-confined and extra prostatic carcinoma compared to nodular prostate hyperplasia (*P* = 0.031 and *P* = 0.004, respectively). Notably, for LOX protein the immunoreactivity score was significantly higher in organ-confined carcinomas compared to nodular prostate hyperplasia (*P* = 0.015). The genotype-phenotype analyses showed higher LOX staining intensity for carriers of the homozygous *LOX* +*473* G-allele (*P* = 0.011). Still, carriers of the *KDR*−604 T-allele were more prone to have higher VEGFR2 expression in prostate epithelial cells (*P* < 0.006).

**Conclusions:**

Protein expression of hypoxia markers (VEGFR2, CAIX and LOX) on prostate epithelial cells was different between malignant and benign prostate disease. Two genetic polymorphisms (*LOX* +473 G > A and *KDR*−604 T > C) were correlated with protein level, accounting for a potential gene-environment effect in the activation of hypoxia-driven pathways in prostate carcinoma. Further research in larger series is warranted to validate present findings.

**Electronic supplementary material:**

The online version of this article (doi:10.1186/s12894-017-0201-y) contains supplementary material, which is available to authorized users.

## Background

Prostate carcinoma is the most common cancer and the second cause of death due to malignancy in men [1]. It is clinically heterogeneous in aggressiveness, not with standing comparable clinicopathological features. Currently, only few biomarkers assist prostate carcinoma risk and aggressiveness prediction [2].

During tumor growth, malignant cells become progressively distant from the vasculature, oxygen supply and nutrients, urging tumor cells to signal to the microenvironment their needs. The hypoxia inducible factor 1 alpha (HIF-1α) is a key factor by which tumors regulate the response to hypoxia, triggering cascades with effects in angiogenesis, energy metabolism, vasomotor function and on apoptosis and proliferation activity [3–5]. In hypoxia, the HIF-1α/HIF-1β complex binds hypoxia response elements in promoters of many downstream target genes, notably vascular endothelial growth factor (*VEGF*), carbonic anhydrase IX (*CAIX*), and lysyl oxidase (*LOX*) promoters. They have been demonstrated to be up-regulated by hypoxia, ensuing aggressive and treatment-resistant tumor phenotypes [3, 5–9]. A large randomized study on radiotherapy and surgical cohorts described that markers of tumor hypoxia and angiogenesis were relevant for localized prostate carcinoma and outcome of radical treatment [10]. However, further studies at the genetic and protein levels are required to confirm molecules in hypoxia pathway as useful markers in prostate carcinoma.

Genetic variants may predispose to prostate carcinoma and influence the clinical outcome [2, 11, 12]. Single nucleotide polymorphisms (SNPs) in genes coding for molecules involved in the response to hypoxia, particularly a functional polymorphism in *HIF1A* gene at locus +1772 C > T [13–20], has been studied in association with prostate carcinoma with controversial results. Current knowledge suggests that we should consider a panel of genes in hypoxia pathway, in order to provide more accurate prediction of the response to tumor hypoxia [21, 22]. Therefore, despite functional SNPs in genes of pathways downstream of HIF-1α, such as *KDR*, *LOX* and *CAIX*, have not been studied so far in prostate carcinoma patients, they merit further research as they represent key molecules in hypoxia-generated stimulus in cancer.

Based on the role of hypoxia-associated molecules in cancer cell biological behaviour and clinical outcome, we assumed there might be an association, at the genetic and protein level, between *HIF1A*, *LOX*, *CA9* and *KDR* genetic variants, the protein expression and prostate carcinoma. Hence, if these polymorphisms modulate protein expression in response to tumor hypoxia, then the knowledge of the genotype could aid identify patients at higher risk for prostate carcinoma and eventually more aggressive disease, thereby making it possible to undertake chemoprevention strategies adjusted to the individual characteristics of the patient.

## Methods

### Patients

Sixty-seven patients with prostate pathology (*n* = 49 with carcinoma, and *n* = 18 with nodular hyperplasia) and elective for prostatic surgery [radical prostatectomy and simple (open) prostatectomy, respectively] at the Porto Hospital Centre - Sto. António Hospital and Porto Military Hospital were included in this study. Inclusion criteria were: 45–75 years of age and for prostate carcinoma absence of previous treatments. Clinicopathological data was collected from clinical files and pathological staging was determined according to European Association of Urology guidelines [23] as organ-confined (T1-T2) (OCPCa) or extra prostatic (T3-T4) (EPCa) disease. Descriptive data is depicted on Table 1. This study was conducted with informed written consent by participants and after approval by the Porto Hospital Centre Ethical Committee.

**Table 1:** Descriptive clinicopathological data of participating patients

	BPH	OCPCa	EPCa
Age at diagnosis, yrs	67.8 ± 8.4	61.3 ± 6.4	63.3 ± 6.3
PSA at diagnosis, ng/mL	5.5 ± 5.1	6.6 ± 2.4	11.9 ± 5.6
Weight of the prostate, g	94.8 ± 32.1	45.9 ± 14.3	56.6 ± 22.7
Gleason Score			
<7	−	14 (43.8)	0 (0.0)
≥7	−	18 (56.3)	19 (100)
Percentage of tumor ^a^, %	−	15.0 (6.3−20.0)	57.0 (28.8−78.8)

Continuous variables were parametric (Shapiro-Wilk) (data presented as mean ± standard deviation) except for percentage of tumor [data shown as median (interquartile range)]. Categorical variable is depicted as number of observations and respective frequencies. *BPH* nodular prostate hyperplasia, *EPCa* extra prostatic cancer, *OCPCa* organ-confined prostate carcinoma, *PSA* prostate specific antigen

^a^ on prostatectomy specimens

### DNA extraction and genotyping

At the time of surgery, a venous blood sample was obtained by forearm venepuncture and the white cell fraction used to extract DNA (QIAmp DNA Blood Mini Kit, Qiagen). Candidate SNPs were selected from the best evidence from published studies that provide information on phenotypic risks. Candidate genes involved in key hypoxia pathways were selected. Four putative functional SNPs in 4 different genes were selected (*HIF1A* +1772 C > T, rs11549465; *CA9* + 201 A > G, rs2071676; *LOX* +473 G > A, rs1800449; *KDR*−604 T > C, rs2071559). These SNPs were genotyped by Real-Time PCR (TaqMan allelic discrimination) using pre-designed validated Taqman assays (Applied Biosystems). Quality control included non-template controls in all runs and blind replicate genotypes assessment in 5% of the samples.

### Immunohistochemistry and scoring

**Fig. 1:**
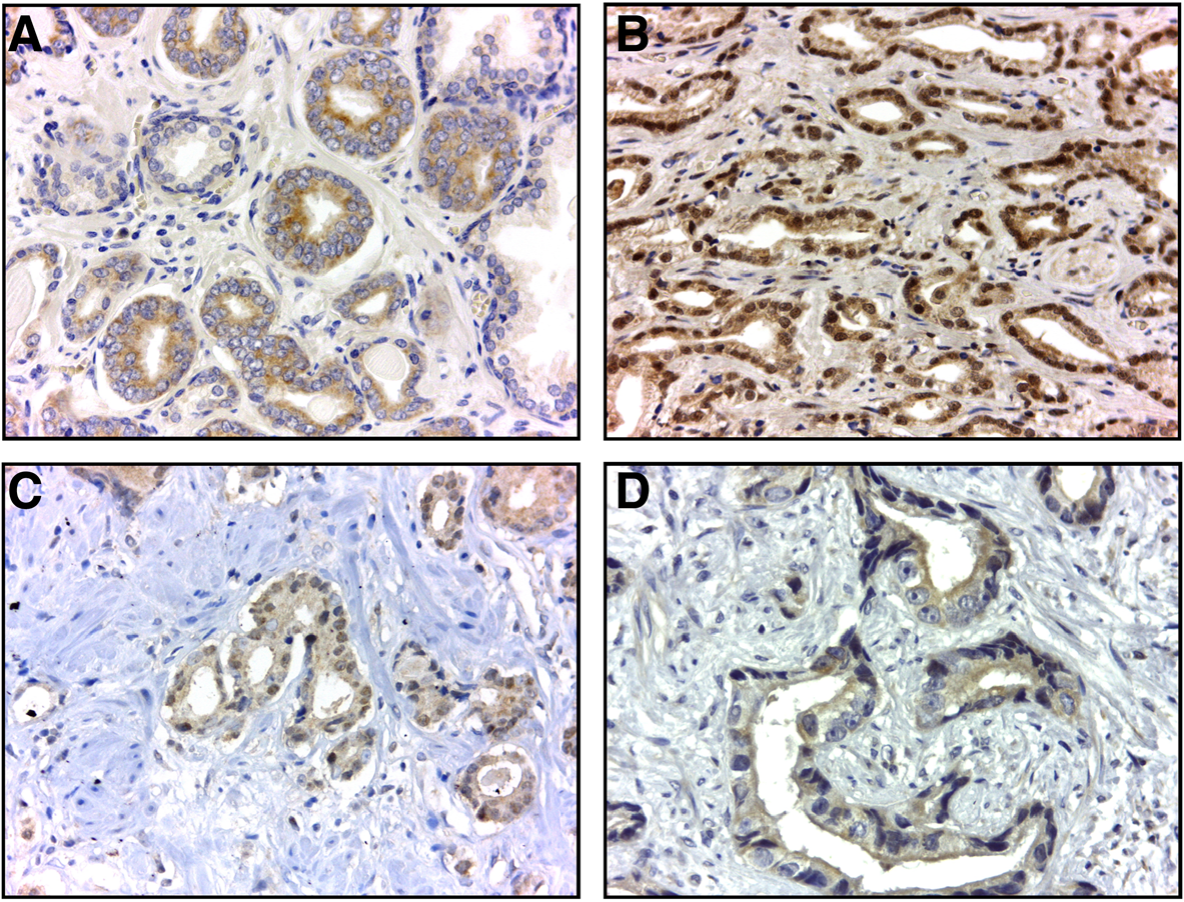
Representative microscopy images of staining for hypoxia markers in prostate tissues (MO, 400×). A) HIF-1α - notice the granular cytoplasmic immunoreactivity of the malignant epithelial cells. In this case, more than 50% of the glands stained. B) LOX - strong and diffuse nuclear immunoreactivity of the epithelial cells. C) CAIX - note a focal apical cytoplasmic immunoreactivity in epithelial cells. D) VEGFR2 - moderate nuclear and weak cytoplasmic expression of the epithelial cells

Formalin–fixed paraffin embedded tissues were morphologically assessed on haematoxylin-eosin stained slides, before tissue microarray construction as previously described [24]. Representative areas of carcinoma and of nodular hyperplasia were selected and included into tissue arrays: prostate carcinoma (*n* = 51) and nodular hyperplasia (*n* = 20), to analyse HIF-1α, LOX, CAIX and VEGFR2 immunohistochemistry expression. Slides were stained with mouse monoclonal antibody to HIF-1α (dilution 1:100, NB100-105, Novus Biologicals), and rabbit polyclonal antibodies to LOX, (dilution 1:100, ab 31238, Abcam), VEGFR2 (dilution 1:200, ab 2349, Abcam) and CAIX, (dilution 1:1000, NB100-417, Novus Biologicals) using the VENTANA BenchMark XT series slide-staining instrument (with the VENTANA ultraView DAB IHC detection kit) (VENTANA, Tucson, AZ, United States). Negative controls omitting the primary antibody confirmed specificity. Immunohistochemistry evaluation was independently reviewed by two pathologists (JRV and AC) to assess VEGFR2 expression in carcinoma vasculature and prostate epithelial cells (carcinoma and nodular hyperplasia), and HIF-1α, LOX and CAIX in prostate epithelial cells (carcinoma and nodular hyperplasia). Discordant cases were discussed in order to attain a final consensus. For VEGFR2 different scoring approaches were evaluated for vessels and epithelial cells as described by Holzer et al. [25], whereas analysis of CAIX, HIF-1α and LOX expression in prostatic epithelial cells (both in carcinoma and nodular hyperplasia) were performed according to Smyth et al. [26], Vergis et al. [10] and Albinger-Hegyi [27], respectively. Briefly, for VEGFR the level of intensity of tumor cell staining (0, no staining; 1+, weak staining; 2+, moderate staining; 3+, intense staining) was made in the cytoplasmic and nuclear compartments simultaneously. The value of each staining level (0, 1, 2 or 3) was multiplied by the respective percentage of tumor cells at that intensity level. A total VEGFR2 H-score represents the sum of the three scores. Regarding LOX, only cytoplasmic immunoreactivity of epithelial cells was considered positive expression, whereas staining in the stromal component was not used. The LOX immunoreactivity score (IRS), was calculated multiplicating the percentage of positive cells (scored 0 if 0% cells; 1 if 1–20% cells; 2 if 21–40% cells; 3 if 41–60% cells; 4 if 61–80%; 5 if 81–100% cells) with staining intensity (with 0 if negative; 1 if weak; 2 if moderate; 3 if strong staining intensity). A representative image of the expression of each aforementioned protein is shown in Fig. 1.

### Statistical analysis

We used means as descriptive statistics for continuous variables and the Shapiro-Wilk test to assess their departure from normality. As appropriate, the Mann–Whitney test and Student *t*-test were used to compare means between prostatic disease groups. The Kruskal Wallis followed by Mann–Whitney two samples tests were used for analyses of non-parametric variables. The Pearson chi-square was used to test for association between categorical variables based on the distribution among diseases, protein expression or genotype groups. Odds ratios (ORs) and 95% confidence intervals (95%CIs) were calculated to evaluate the associations between CAIX expression with risk for developing organ-confined and extra prostatic carcinoma. When appropriate, non-parametric Spearman’s correlation was computed to assess the statistical dependence between variables. Analyses were performed using SPSS 17.0. The datasets analysed during the current study are available from the corresponding author on reasonable request.

## Results

### Association of hypoxia proteins with prostate cancer and extra-prostatic disease

**Fig. 2:**
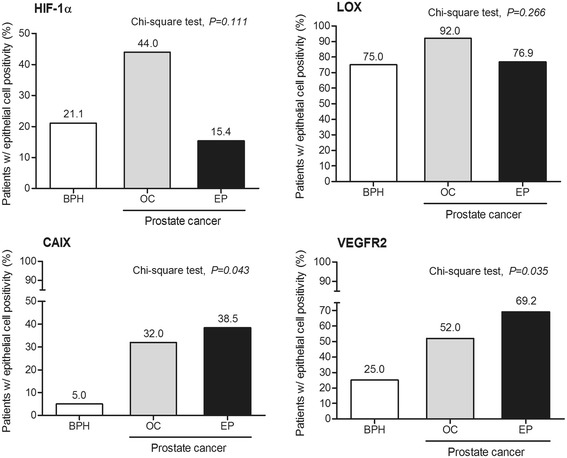
Frequency of patients with positive staining in benign (BPH) and malignant (organ-confined and extra prostatic disease) epithelial cells. CAIX, carbonic anhydrase IX; HIF-1α, hypoxia inducible factor - 1 alpha; LOX, lysyl oxidase; VEGFR2, vascular endothelial growth factor receptor 2. BPH, nodular prostate hyperplasia; EP, extra prostatic disease; OC, organ-confined disease

To assess the prevalence of the key hypoxia-associated proteins in prostate carcinomas and nodular prostate hyperplasia, a tissue microarray was constructed for immunohistochemistry analyses. Immunohistochemistry for cytoplasmic HIF-1α demonstrated a non-significant trend (*P* = 0.111) for increased proportion of localized prostate carcinoma patients with positive malignant prostatic epithelial cells (Fig. 2). CAIX immunoreactivity was observed in the cytoplasm of epithelial cells and significant differences were found among disease groups: CAIX expression was predominantly positive in epithelial cells of carcinomas (*P* = 0.043) (Fig. 2).

**Fig. 3:**
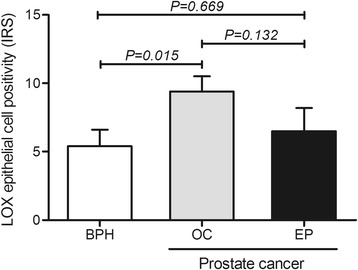
Comparison of LOX immunoreactivity score in prostate epithelial cells of benign and malignant patients. BPH, nodular prostate hyperplasia; EP, extra prostatic disease; OC, organ-confined disease. LOX, lysyl oxidase; IRS, immunoreactivity score. Kruskall-Wallis followed by Mann–Whitney non-parametric tests were used to calculate differences between prostatic pathologies

**Fig. 4:**
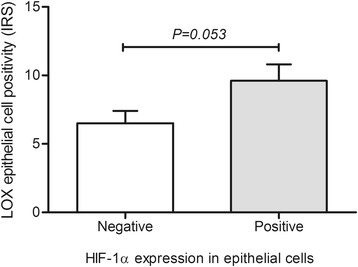
LOX immunoreactivity score by HIF-1α positivity in epithelial cells. Patients with positive HIF-1α expression are prone to higher LOX IRS. HIF-1α, hypoxia inducible factor – 1 alpha; LOX, lysyl oxidase. IRS, immunoreactivity score. Mann–Whitney non-parametric test was used to calculate differences between positive and negative HIF-1α expression

Lysyl oxidase protein expression was found in prostate epithelial cells of a high percentage of cases, notably in carcinomas of patients with organ-confined malignancy (92.0%), but no significant differences were found among pathologic groups (*P* = 0.266) (Fig. 2). Nevertheless, the immunoreactivity score (IRS), which combines intensity with amount of cells positive for LOX in prostate epithelial cells, was significantly higher in organ-confined carcinomas compared to nodular prostate hyperplasia (*P* = 0.015) (Fig. 3). Noteworthy, patients with positive HIF-1α expression were more prone to have higher immunoreactivity score for LOX (*P* = 0.053) (Fig. 4). In addition, a trend exists for HIF-1α immunostaining grade to be correlated with LOX IRS expression (Spearman correlation coefficient, *r* ^2^ = 0.255, *P* = 0.055).

**Fig. 5:**
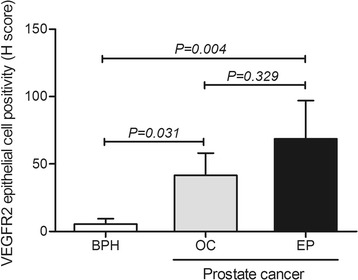
Expression of VEGFR2 (H score) in prostate epithelial cells according to prostatic diseases. BPH, nodular prostate hyperplasia; EP, extra prostatic disease; OC, organ-confined disease. VEGFR2, vascular endothelial growth factor receptor 2. Kruskall-Wallis followed by Mann–Whitney non-parametric tests were used to calculate differences between prostatic pathologies

Cytoplasmic and nuclear VEGFR2 immunoreactivity was observed in vascular endothelial cells of approximately 20% of all samples. The difference between vascular positivity for VEGFR2 in nodular prostate hyperplasia and both organ-confined and extra prostatic carcinomas was not statistically significant (*P* = 0.971). As for VEGFR2 staining in epithelial prostate cells, almost 70% of patients with extra prostatic carcinomas and approximately half of organ-confined carcinomas showed tumor cell immunoreactivity for VEGFR2, whereas only 25% of nodular prostate hyperplasia were positive (*P* = 0.035) (Fig. 2). The VEGFR2 expression scores in the prostate epithelial cells in nodular prostate hyperplasia (5.6 ± 3.9) compare to either organ-confined (41.6 ± 16.5) or extra prostatic carcinomas (68.7 ± 28.4) were statistically different (*P* = 0.031 and *P* = 0.004, respectively) (Fig. 5). The VEGFR2 epithelial cell H-score for samples that were positive for VEGFR2 in the vasculature showed a trend for being higher than those with negative immunoreactivity status (*P* = 0.062), indicating a positive association between the expression of VEGFR2 in the prostatic epithelial cells and the vasculature.

### Genotype-phenotype correlation

The genotypic distribution in polymorphisms *HIF1A* +1772 C > T, *LOX* +473 G > A, *CA9* + 201 A > G and *KDR*−604 T > C is shown in Additional file 1: Table S1. There was no over-represented genotype in disease groups using either the additive or recessive models.

**Fig. 6:**
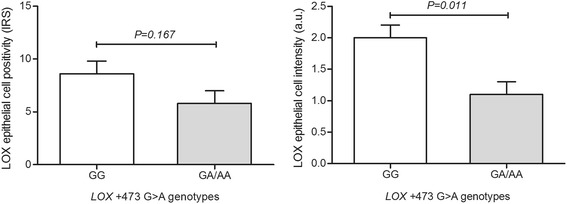
LOX protein expression (both for immunoreactivity score and staining intensity) according to *LOX* +473 G > A polymorphism. IRS, immunoreactivity score; *LOX*, lysy oxidase; a.u., arbitrary units

**Fig. 7:**
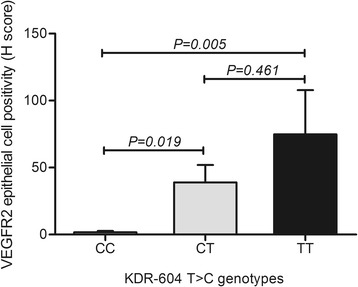
VEGFR2 protein expression (H score) according to *KDR*−604 T > C polymorphism. *KDR*, gene coding for VEGFR2 protein; VEGFR2, vascular endothelial growth factor receptor 2

There was lack of association between both *HIF1A* +1772 C > T and *CA9* + 201 A > G genotypes and positivity or intensity for HIF-1α and CAIX protein expression (Table 2). Conversely, the LOX immunoreactivity intensity was significantly higher in individuals carrying the *LOX* +*473* homozygous G allele (GG, 2.0 ± 0.2) compare to A carriers (1.1 ± 0.2) (*P* = 0.011) (Fig. 6), despite no significance was achieved for IRS (but with similar trend) according to *LOX* genotypes in recessive model. Patients with at least one *KDR*−604 T-allele were more prone to have VEGFR2 expression in prostate epithelial cells but not in vessels (Table 3). Since the presence of VEGFR2 immunoreactivity in epithelial cells, but not in vessels, was associated with the *KDR* genetic polymorphism, we looked for its association with VEGFR2 H-score only in prostate epithelial cells. The H-score was significantly higher in cases carrying the T allele (CT, 38.9 ± 13.0 and TT, 74.7 ± 33.0) compare to homozygous C (1.64 ± 1.0) (Fig. 7). Both additive and recessive models show that the allele T was related with increased VEGFR2 epithelial cell positivity (*P* = 0.017 and *P* = 0.006, respectively).

**Table 2:** Association of the genetic polymorphisms in *HIF1A* +1772 C > T and *CA9* + 201 A > G with HIF-1α and CAIX immunoreactivity in prostatic epithelial cells

	Recessive model (*HIF1A* and *CA9*)		
*HIF*-*1*α *expression*	*CC*	*TT*/*CT*	P ^a^
Negative	28 (0.76)	9 (0.24)	
Positive	10 (0.77)	3 (0.23)	0.928
<50%	32 (0.74)	11 (0.26)	
≥50%	6 (0.86)	1 (0.14)	0.516
CAIX expression	GG	GA/AA	
Negative	9 (0.75)	20 (0.69)	
Positive	3 (0.25)	9 (0.31)	0.699

^a^ Fisher exact test

**Table 3:** Association of the *KDR*-*604 T* > *C* genetic polymorphism with VEGFR2 immunoreactivity in vessels and in prostatic epithelial cells

	Additive model				Recessive model		
	CC	CT	TT	P ^a^	CC	TT/CT	P ^a^
Vessels VEGFR+							
Negative	11 (0.26)	22 (0.53)	9 (0.21)		11 (0.26)	31 (0.78)	
Positive	3 (0.25)	5 (0.42)	4 (0.33)	0.681	3 (0.25)	9 (0.22)	0.626
Epithelial cells VEGFR+							
Negative	11 (0.39)	13 (0.47)	4 (0.14)		11 (0.39)	17 (0.42)	
Positive	3 (0.11)	14 (0.54)	9 (0.35)	0.039	3 (0.11)	23 (0.58)	0.030

^a^ Fisher exact test

Only data from prostate carcinomas was used to evaluate if hypoxia proteins associated with Gleason score or prostate specific antigen (PSA) > 10 ng/mL (Table 4). Trends were observed for higher VEGFR2 H-score expression in more undifferentiated carcinomas (Gleason ≥7) (*P* = 0.099) and in patients with PSA ≥ 10 ng/mL (*P* = 0.085), and for positive CAIX expression in prostate carcinomas from patients with PSA above 10 ng/mL (*P* = 0.078).

**Table 4:** Expression of proteins from hypoxia pathways in prostate cancer patients, by Gleason grade and PSA value

	Gleason grade (*n* = 38)			PSA at diagnosis (*n* = 36)		
	<7	≥7	P	<10	≥10	P
VEGFR2 H-score^a^	30.9 ± 24.7	60.1 ± 17.9	0.099	30.2 ± 1.2	80.0 ± 33.5	0.085
LOX immunoreactivity score^a^	10.2 ± 1.6	7.6 ± 1.1	0.184	9.2 ± 1.1	6.6 ± 1.8	0.242
HIF-1α expression^b^						
Negative	6 (0.50)	19 (0.73)		17 (0.65)	8 (0.80)	0.335^c^
Positive	6 (0.50)	7 (0.27)	0.163	9 (0.35)	2 (0.20)	
CAIX expression^b^						
Negative	10 (0.83)	15 (0.58)		19 (0.73)	5 (0.50)	
Positive	2 (0.17)	11 (0.42)	0.117 ^c^	7 (0.27)	5 (0.50)	0.078

*PSA* prostate specific antigen, *VEGFR*2 vascular endothelial growth factor receptor 2, *LOX* lysyl oxidase, *HIF*1*a* hypoxia inducible factor 1 alpha, *CAIX* carbonic anhydrase IX

^a^ Kruskal Wallis and Mann–Whitney U tests for VEGFR2 H-score in epithelial cells; ^b^ Chi-square test.^c^ Fisher exact test

## Discussion

Tumor-associated hypoxia was found in over 70% of solid malignancies, including prostate carcinoma [3]. It promotes tumor progression and resistance to therapies through an effect in reducing apoptosis, and increasing tumor cell proliferation and neoangiogenesis [5]. However, the hypoxia-driven HIF-1α upregulation also activates downstream pathways involved in metabolism (e.g. CAIX), angiogenesis (e.g. VEGF/VEGFR2 pathway) and extracellular matrix activity (e.g. LOX), which can modulate cancer behavior [28].

Experimental studies with prostate cancer cells demonstrated that HIF-1α overexpression was associated with higher proliferation and metastatic potential [29]. Likewise, a greater expression of HIF-1α has been found in human prostate carcinomas compared to nodular prostate hyperplasia [30, 31]. For prostate carcinoma and other oncologic models, besides the observed higher amount of HIF-1α in tumors, increased HIF-1α expression was also associated with prognosis [10, 32–35]. In the current study, we found a trend for higher HIF-1α protein expression in prostate carcinomas compared to nodular prostate hyperplasia, which may be explained by the limited samples analysed. The use of cytoplasmic rather than nuclear staining, is unlikely to have influenced our results, since this method has been published before, reporting positive associations of HIF-1α with prostate carcinoma and prognosis [10, 30].

Albeit mainly distributed in vascular endothelial cells, also epithelial cells express VEGFR2 that signals through signal transducer and activator of transcription 3 (STAT3), mitogen-activated protein kinase (MAPK) or phosphoinositide-3-kinase (PI3K) intracellular signalling cascades [36–38]. Unambiguously, the VEGFR2 was shown to regulate protein kinase B (Akt)/mammalian target of rapamycin (mTOR)/ribosomal protein S6 kinase beta-1 (P70S6K) signalling pathway in PC-3 prostate cancer cell line [39]. In the present study, VEGFR2 was more frequently expressed in epithelial tumor cells of organ confined or extra prostatic carcinomas than in nodular prostate hyperplasia, and to lower extent in endothelial cells. Hence, at least in prostate tissue, VEGFR2 expression is not specific of endothelial cells; it is mainly expressed in malignant epithelium where VEGF can act as a promoter of tumor cell proliferation. The expression of VEGFR2 in epithelial prostate carcinoma cells has been rarely reported, and its role in the occurrence and development of prostate cancer remains unclear. Previous immunohistochemistry studies reported VEGFR2 expression in high-grade prostate intra-epithelial neoplasia and carcinomas of the prostate [40–42], whereas gene expression findings evidenced expression of *KDR* mRNA in prostate cancer cell lines and a functional impact of using a *KDR* antisense oligonucleotide in suppressing cell proliferation and promoting apoptosis [43, 44].

The body of past evidences, taken together with present findings indicates that the distribution of VEGFR2 expression towards epithelial prostate carcinoma cells supports a function for VEGF that is not limited to angiogenesis. Thus, abrogation of VEGFR2 signalling in malignant epithelial cells may prove an effective therapeutic modality for the treatment of prostate cancer. At present, two anti-angiogenic drugs are being tested in the phase III setting for men with prostate cancer, carbozantinib (a dual VEGFR2/MET inhibitor) and tasquinimod (down-regulator of HIF-1α), which previously showed beneficial and encouraging results on phase II trials [45].

Cancer-associated hypoxia switches cell metabolism towards increased production of acidic metabolites. However, tumor cells have to adapt to hypoxia and acidosis in order to survive. CAIX is a membrane-bound protein crucial to a wide variety of processes, including pH regulation in the highly metabolically active malignant cells. Expression of CAIX is associated with tumor cell hypoxia in a variety of human tumors, including urologic cancers [46–49]. Carbonic anhydrase IX gene (*CA9*) is a target of HIF-1α that is up-regulated in response to hypoxia [50]. The expression of CAIX in prostate carcinoma has been rarely reported. *CA9* mRNA expression increases reliably following hypoxia incubation of PC-3 cells [51], although no significant differences in *CA9* mRNA expression were found when comparing nodular prostate hyperplasia with prostate carcinomas [7]. However, other studies reported lack of CAIX expression in primary prostate carcinoma and hypothesized that alternative pathway for maintaining pH balance (e.g. monocarboxylate transporters 2 and 4) [26, 52, 53] may be more relevant than CAIX.

Our results disclosed increased frequency of cases with epithelial cell positivity for CAIX expressing in organ confined and extra prostatic carcinomas compared to BPH. Despite recent concern arisen for the specificity of the CAIX polyclonal antibody generated against a C-terminal peptide in detecting CAIX (except when used at high dilution, in prostate tissues) [54], in this study we used the antibody at a dilution of 1:1000 and found membrane-bound staining for CAIX. Therefore, our findings are likely to reflect reliable expression of CAIX in epithelial prostate cells. Our findings taken together with reports of CAIX expression in malignant prostate epithelial cells [7, 51, 55] sustains the need for reconsidering CAIX role in prostate carcinoma. CAIX may serve as one of the mechanisms by which prostate carcinoma cells regulate extracellular pH and induce cytoplasmic alkalization.

The lysyl oxidase gene (*LOX*), one of the overexpressed genes among a tumor hypoxia signature [56, 57], was shown to be directly regulated by HIF-1α transcription factor and is essential for hypoxia-induced metastasis and cancer cell proliferation [58]. Hypoxia-driven cancer cell invasion is severely impaired when LOX expression or oxidase activity were inhibited [59]. In prostate tissue we found that the LOX immunoreactivity score correlated with HIF-1α expression, thus supporting the regulatory nature of HIF-1α in LOX expression. Furthermore, although we have not observed an overrepresentation of cases with positive LOX expression in carcinomas compared to nodular prostate hyperplasia, the LOX immunoreactivity score was significantly higher in organ confined prostate carcinomas compared to nodular prostate hyperplasia. Interestingly, previous reports showed significantly increased expression of *LOX* mRNA in prostate carcinomas compared to nodular prostate hyperplasia [7], whereas stronger LOX expression was also observed in other solid malignancies [27, 60, 61]. LOX is known to participate in critical biological functions that include cell migration, cell polarity, epithelial-to-mesenchymal transition (EMT) and angiogenesis [58] (reviewed in Fraga et al., 2015) [62], which fits with the increased LOX expression found in our carcinomas. Altogether, we suggest the possibility that a HIF-1α/LOX regulatory mechanism may act in synergy to foster tumor formation along with the adaptation of tumor cells to hypoxia.

The analysis of protein expression in distinct pathological groups (by stage, differentiation score and PSA serum levels at diagnosis), which are predictive of prostate cancer aggressiveness, showed at most only trends for increased expression of VEGFR2 in carcinomas with Gleason >7 or patients with PSA > 10 ng/mL, and of CAIX in patients with PSA > 10 ng/mL. These findings indicate relevant clues but require further studies.

The genotypic distributions for the putative functional target SNPs in *HIF1A*, *LOX*, *CA9* and *KDR* were similar between nodular prostate hyperplasia and prostate carcinomas. We might have hypothesized that carriers of variant alleles are prone to be more susceptible to have cancer, but the underpowered sample size limits conclusions regarding genetic association for these SNPs. Nevertheless, it is expected that only the combination of several SNPs within pathways or mechanisms may have significant impact in the association with complex diseases as prostate carcinoma. Further studies are warranted to evaluate the predictive/prognostic value of these genetic polymorphisms in prostate cancer.

In this study, evaluation of protein expression according to SNPs in the respective coding genes disclosed a genotype-phenotype effect for the *LOX* and *KDR* SNPs, but no functional validation at the protein level was observed for the studied *HIF1A* and *CA9* SNPs. In the *HIF1A* gene, a C-to-T substitution at locus +1772 (rs11549465) results in non-synonymous proline-by-serine aminoacid substitution at codon 582. Association studies of this SNP with prostate carcinoma risk and with microvessel density, yielded conflicting results [13, 16, 19, 20, 63–65]. This SNP localizes in the oxygen-dependent domain of the gene where the variant allele was shown to stabilize *HIF1A* mRNA and enhance *HIF1A* transcriptional activity [64]. In our study there were no differences in HIF-1α protein expression according to the *HIF1A* +1772 C > T genotypes as reported previously in localised prostatic carcinomas [16]. As we measured HIF-1α protein levels and it is known that *HIF1A* is subjected to post-transcriptional and post-translational regulation [66], this SNP may indeed influence mRNA transcription that is not reflected in protein expression. The low frequency of T homozygous genotype in our sample (only 2 cases carried TT genotype) may have influenced statistical power, since the HIF-1α protein and mRNA overexpression have been associated with the *HIF1A* +1772 TT [14, 67, 68].

A functional genetic variant on *KDR* gene that codifies for VEGFR2 is located in the promoter region (−604, rs2071559), where a T-to-C substitution occurs. Preceding in vitro luciferase assays showed that the C-allele was associated with lower transcription activity than T-allele, whereas serum VEGFR2 levels were significantly lower in CC versus TT carriers [69]. Interestingly, we found that CT and TT carriers had significantly increased VEGFR2 expression in prostate epithelial cells. We postulate that this SNP might prove useful for predictive and/or prognostic evaluations in prostate carcinoma. Studies in colorectal cancer reported association of this SNP in *KDR* with susceptibility and recurrence [70, 71], whereas, to the best of our knowledge, no studies using this SNP were conducted in prostate carcinoma patients. Likewise, it is expected that this SNP might increase susceptibility to prostate cancer by upregulating the number of available VEGFR2 proteins in malignant cells.

A SNP in exon 1 of *CA9* gene is located at locus +201 (rs2071676), where an A-to-G substitution leads to a change of valine-by-methionine in codon 33. Although we observed an overrepresentation of CAIX positive immunoreactivity in prostate carcinoma compared to BPH, the nonsynonymous SNP in *CA9* + 201 were unable to explain variations in the levels of CAIX protein expression in the prostatic tissue. Likewise, a recent report described lack of association between the *CA9* + 201 SNP with CAIX protein expression in renal cell carcinoma [72]. These findings may suggest that lack of influence of this SNP in protein expression, even though the potential molecular structure modifications of this nonsynonymous substitution (valine to methionine) in CAIX protein activity remains to be confirmed. In fact, genetic association studies that included the *CA9* + 201 A > G polymorphism showed neither risk for renal cell carcinoma [72] nor for oral squamous cell carcinoma [73]. Noteworthy, the G-allele was associated with lymph node metastasis in oral cancer and represented increased risk for cancer when combined into a haplotype with other two SNPs in this gene [73]. Furthermore, another SNP in *CA9* (rs12553173) was independently associated with improved overall survival and greater likelihood of response to therapy in renal cell carcinoma [72], thus warranting further functional analysis. In our study, although we are aware that haplotype analyses can be expedite over analysis of individual SNPs for detecting an association between alleles and a disease phenotype, the small size sample prevented the consideration of such evaluation.

The *LOX* gene is translated and secreted as a proenzyme (Pro-LOX), and then processed to a functional enzyme (LOX) and a propeptide (LOX-PP) [74, 75]. While LOX-PP was described as a Ras tumor suppressor, reversing mesenchymal tumor cells to a more epithelial phenotype [76–78], the LOX enzyme was found to facilitate a more migratory and invasive phenotype during breast cancer progression [58, 79]. We studied a SNP in *LOX* gene that has been identified at locus +473 (rs1800449), presenting a G-to-A substitution that cause an aminoacid substitution arginine-by-glutamine in codon 158. This SNP located in a highly conserved region within LOX-PP has been associated with attenuated ability of LOX-PP to oppose the effects of LOX, resulting in tumor cell invasive phenotype. Functional studies revealed that the A-allele decreases the protective capacity of LOX-PP, while increasing the Pro-LOX-associated invasive ability of tumor cells [78]. When evaluating LOX immunoreactivity and expression intensity by immunohistochemistry in prostate tissues, we found it significantly lower in carriers of the *LOX* +473 A-allele. Indeed, *LOX* A-carriers disclosed decreased LOX protein expression in the nucleus of prostate epithelial cells.

The complex nature of LOX protein domain structure and biological functions makes noticeable that it can act as both a tumor suppressor and a metastasis promoter gene in cancer [80]. Under hypoxic conditions, the increased expression of LOX enzyme correlates with tumor invasiveness [81, 82]. In the present study, we found that lysyl oxidase was present primarily intracellular in the nucleus of epithelial cells, which fits with other reports asserting that this enzyme may have important functions in secretory cells, either as catalyser of histones in the nucleus or in association with cytoskeletal proteins at the cytoplasm [83, 84]. Thus, our findings seem to suggest a wider variety of functions for LOX in prostate epithelial cells, beyond those related to cross-link formation in collagen and elastin, which merit further research. We hypothesize that the trafficking of LOX towards inside the cell or a specific cell compartment may be subordinated to the structural molecular characteristics and folding of the protein, which could be determined by *LOX* +473 G > A polymorphism. Further studies should clarify the meaning of increased nuclear LOX intensity for PCa development.

Our endeavour to study the genotype-phenotype correlation in key hypoxia markers and its association with prostate cancer yielded novel and interesting findings, nevertheless our results should be interpreted in the context of several potential limitations. Sample size was a major issue as conclusions were impracticable for genetic association analysis and limited for genotype-phenotype inferences. Nevertheless, considering the hypothesis-generating nature of this study, we report findings that provide important clues to further work in larger samples. The use of tissue microarrays for immunohistochemical evaluation has been subject of concern mainly due to limited sample of diagnostic tissue, although in our series the representative tumor sections were adequately selected by an experienced pathologist. The comparison of hypoxia markers between patients with benign and malignant prostate disease might attenuate differences since it is known that hypoxia is altered in cancer but also in benign hyperproliferative diseases. The group of benign prostate disease seemed adequate for several order of reasons: 1) the diagnosis was contemporary with that of cancers; 2) their advanced age at diagnosis allowed matching with elderly prostate cancer patients; 3) all patients underwent digital rectal examination, PSA testing and prostate needle biopsy, making the possibility of crossover remote, and 4) most men develop nodular prostate hyperplasia or chronic prostatitis by the 7th–8th decades of life, making it normal in men of that age to carry benign prostatic disease.

## Conclusions

Prostate carcinoma triggers an increase in hypoxia, which regulates *HIF1A* that in turn impacts downstream the expression of LOX, CAIX and VEGFR2 in tumor cells. In this study we observed that the inherited genetic variants in *LOX* and *KDR* seem to modulate the expression of LOX and VEGFR2 in carcinoma cells, supporting a gene-tumor microenvironment interaction in the activation of hypoxia-driven pathways in prostate carcinoma. Results presented here warrant further research in larger samples in order to evaluate the predictive and prognostic value of *KDR* and *LOX* SNPs in prostate carcinoma.

## Additional file

Additional file 1: Table S1. Genotypic distribution of functional SNPs in genes of hypoxia pathways by disease status using additive and recessive models analyses. The genotypic distribution of studied SNPs in genes of hypoxia pathways, using additive and recessive models, are shown according to disease status. (DOCX 17 kb)

## Abbreviations

95%CI: 95% confidence interval
Akt: Protein kinase B
BPH: Nodular prostate hyperplasia
CA9: Carbonic anhydrase IX gene
CAIX: Carbonic anhydrase IX
EMT: Epithelial-to-mesenchymal transition
EPCa: Extra prostatic (T3–T4) Prostate Cancer
HIF-1α: Hypoxia-inducible factor 1 alpha
HIF-1β: Hypoxia-inducible factor 1 beta
HIF1A: HIF-1α gene
IRS: Immunoreactivity score
KDR: VEGFR2 gene
LOX: Lysyl oxidase
LOX-PP: Lysyl oxidase propeptide
MAPK: Mitogen-activated protein kinase
mTOR: Mammalian target of rapamycin
OCPCa: Organ-confined (T1–T2) Prostate Cancer
OR: Odds ratio
P70S6K: Ribosomal protein S6 kinase beta-1
PCR: Polymerase chain reaction
PI3K: Phosphoinositide-3-kinase
PSA: Prostate specific antigen
SNP: Single nucleotide polymorphisms
STAT3: Signal transducer and activator of transcription 3
VEGF: Vascular endothelial growth factor
VEGFR2: Vascular endothelial growth factor receptor 2
