# Supplementary material for: Genetic polymorphisms in key hypoxia-regulated downstream molecules and phenotypic correlation in prostate cancer
Source: BMC Urol. 2017 Jan 31;17:12. doi: 10.1186/s12894-017-0201-y (PMC5282787; doi:10.1186/s12894-017-0201-y)
Supplement: Additional file 1: Table S1. — Genotypic distribution of functional SNPs in genes of hypoxia pathways by disease status using additive and recessive models analyses. The genotypic distribution of studied SNPs in genes of hypoxia pathways, using additive and recessive models, are shown according to disease status. (DOCX 17 kb) [file 12894_2017_201_MOESM1_ESM.docx]

Supplementary table 1. Genotypic distribution of functional SNPs in genes of hypoxia pathways by disease status using additive and recessive models analyses

|  | Prostatic disease status | | |  |
| --- | --- | --- | --- | --- |
| *HIF1A* +1772 C>T genotypes | BPH | OCPCa | EPCa | P * |
| *Additive model* |  |  |  |  |
| CC | 10 (0.59) | 23 (0.82) | 14 (0.78) |  |
| CT | 5 (0.29) | 5 (0.18) | 4 (0.22) |  |
| TT | 2 (0.12) | 0 (0.0) | 0 (0.0) | 0.144 |
| *Recessive model* |  |  |  |  |
| CC | 10 (0.59) | 23 (0.82) | 14 (0.78) |  |
| TT/CT | 7 (0.41) | 5 (0.18) | 4 (0.22) | 0.205 |
| *LOX* +473 G>A genotypes |  | | |  |
| *Additive model* |  |  |  |  |
| GG | 6 (0.71) | 16 (0.55) | 13 (0.72) |  |
| GA | 2 (0.29) | 11 (0.38) | 4 (0.22) |  |
| AA | 0 (0.0) | 2 (0.07) | 1 (0.06) | 0.740 |
| *Recessive model* |  |  |  |  |
| GG | 6 (0.71) | 16 (0.55) | 13 (0.72) |  |
| AA/GA | 2 (0.29) | 13 (0.45) | 5 (0.28) | 0.442 |
| *CA9* +201 A>G genotypes |  |  |  |  |
| *Additive model* |  |  |  |  |
| GG | 3 (0.38) | 9 (0.31) | 5 (0.29) |  |
| GA | 5 (0.62) | 18 (0.62) | 10 (0.59) |  |
| AA | 0 (0.0) | 2 (0.07) | 2 (0.12) | 0.882 |
| *Recessive model* |  |  |  |  |
| GG | 3 (0.38) | 9 (0.31) | 5 (0.29) |  |
| GA/AA | 5 (0.62) | 20 (0.69) | 12 (0.71) | 0.918 |
| *KDR -604 T>C* genotypes |  |  |  |  |
| *Additive model* |  |  |  |  |
| CC | 6 (0.33) | 8 (0.26) | 3 (0.17) |  |
| CT | 8 (0.45) | 15 (0.48) | 13 (0.72) |  |
| TT | 4 (0.22) | 8 (0.26) | 2 (0.11) | 0.436 |
| *Recessive model* |  |  |  |  |
| CC | 6 (0.33) | 8 (0.26) | 3 (0.17) |  |
| TT/CT | 12 (0.67) | 23 (0.74) | 15 (0.83) | 0.515 |

* Fisher exact test. BPH, nodular prostate hyperplasia; OCPCa, organ-confined prostate carcinoma; EPCa, extra prostatic carcinoma. *CA9*, carbonic anhydrase IX gene; *HIF1A*, hypoxia inducible factor 1 alpha gene; *KDR*, vascular endothelial growth factor receptor 2 gene; *LOX*, lysyl oxidase gene.
